# Targeting mitochondrial energetics reverses panobinostat‐ and marizomib‐induced resistance in pediatric and adult high‐grade gliomas

**DOI:** 10.1002/1878-0261.13427

**Published:** 2023-05-12

**Authors:** Esther P. Jane, Matthew C. Reslink, Taylor A. Gatesman, Matthew E. Halbert, Tracy A. Miller, Brian J. Golbourn, Stephanie M. Casillo, Steven J. Mullett, Stacy G. Wendell, Udochukwu Obodo, Dinesh Mohanakrishnan, Riya Dange, Antony Michealraj, Charles Brenner, Sameer Agnihotri, Daniel R. Premkumar, Ian F. Pollack

**Affiliations:** ^1^ Department of Neurosurgery University of Pittsburgh School of Medicine PA USA; ^2^ John G. Rangos Sr. Research Center Children's Hospital of Pittsburgh PA USA; ^3^ Department of Pharmacology and Chemical Biology University of Pittsburgh PA USA; ^4^ Department of Diabetes & Cancer Metabolism City of Hope Medical Center Duarte CA USA; ^5^ UPMC Hillman Cancer Center Pittsburgh PA USA

**Keywords:** glioma, marizomib, mitochondria, OXPHOS, panobinostat, resistance

## Abstract

In previous studies, we demonstrated that panobinostat, a histone deacetylase inhibitor, and bortezomib, a proteasomal inhibitor, displayed synergistic therapeutic activity against pediatric and adult high‐grade gliomas. Despite the remarkable initial response to this combination, resistance emerged. Here, in this study, we aimed to investigate the molecular mechanisms underlying the anticancer effects of panobinostat and marizomib, a brain‐penetrant proteasomal inhibitor, and the potential for exploitable vulnerabilities associated with acquired resistance. RNA sequencing followed by gene set enrichment analysis (GSEA) was employed to compare the molecular signatures enriched in resistant compared with drug‐naïve cells. The levels of adenosine 5′‐triphosphate (ATP), nicotinamide adenine dinucleotide (NAD)^+^ content, hexokinase activity, and tricarboxylic acid (TCA) cycle metabolites required for oxidative phosphorylation to meet their bioenergetic needs were analyzed. Here, we report that panobinostat and marizomib significantly depleted ATP and NAD^+^ content, increased mitochondrial permeability and reactive oxygen species generation, and promoted apoptosis in pediatric and adult glioma cell lines at initial treatment. However, resistant cells exhibited increased levels of TCA cycle metabolites, which required for oxidative phosphorylation to meet their bioenergetic needs. Therefore, we targeted glycolysis and the electron transport chain (ETC) with small molecule inhibitors, which displayed substantial efficacy, suggesting that resistant cell survival is dependent on glycolytic and ETC complexes. To verify these observations *in vivo*, lonidamine, an inhibitor of glycolysis and mitochondrial function, was chosen. We produced two diffuse intrinsic pontine glioma (DIPG) models, and lonidamine treatment significantly increased median survival in both models, with particularly dramatic effects in panobinostat‐ and marizomib‐resistant cells. These data provide new insights into mechanisms of treatment resistance in gliomas.

## Graphical abstract

Brain‐penetrant panobinostat, a histone deacetylase inhibitor, and proteasomal inhibitor marizomib displayed synergistic activity against pediatric and adult high‐grade gliomas. Despite the remarkable initial response to this combination, resistance emerged. The resistant cells exhibited increased levels of TCA cycle metabolites, required to meet their bioenergetic needs. Therefore, we targeted the electron transport chain with small‐molecule inhibitors, which displayed substantial efficacy.

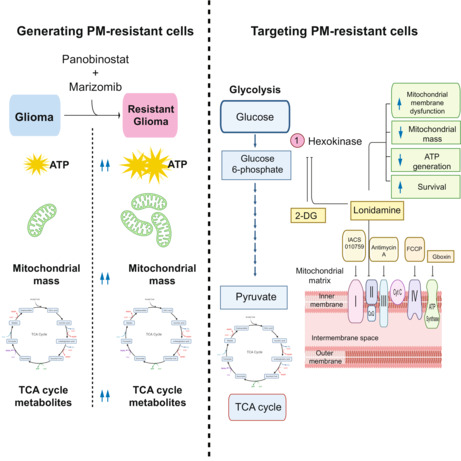

**Abbreviations**
2‐DG: 2‐deoxy‐d‐glucose
ATP: adenosine 5′‐triphosphate
BSA: bovine serum albumin
cADPR: cyclic (ADP‐ribose)
CCCP: carbonyl cyanide m‐chlorophenyl hydrazone
CNS: central nervous system
DIPG: diffuse intrinsic pontine glioma
DMG: diffuse midline glioma
DMSO: dimethyl sulfoxide
FACS: fluorescence‐activated cell sorting
FCCP: carbonyl cyanide 4‐(trifluoromethoxy)phenylhydrazone
FDR: false discovery rate
FITC: fluorescein isothiocyanate
GBM: glioblastoma
H2DCFH‐DA: 2′,7′‐dichlorodihydrofluorescein diacetate
HDAC: histone deacetylase
HDACi: histone deacetylase inhibitor
HE: hydroethidine
HGG: high‐grade glioma
NaAD: nicotinic acid adenine dinucleotide
NAC: acetylcysteine *N*‐acetyl‐l‐cysteine
NAD: nicotinamide adenine dinucleotide
NaMN: nicotinic acid mononucleotide
NAO: 10‐*N*‐nonyl‐acridine orange
NMNAT: nicotinamide mononucleotide adenylyl transferase
OXPHOS: oxidative phosphorylation
PAGE: polyacrylamide gel electrophoresis
PBS: phosphate‐buffered saline
PDH: pyruvate dehydrogenase
PI: propidium iodide
PM‐resistant: panobinostat‐ and marizomib‐resistant
QPRT: quinolinic acid phosphoribosyltransferase
ROS: reactive oxygen species
SDS/PAGE: sodium dodecyl sulfate‐polyacrylamide gel electrophoresis
TCA: tricarboxylic acid
WT: wild‐type

## 1 Introduction

Malignant gliomas are the most common and fatal central nervous system tumors affecting both adults and children [1, 2]. High‐grade gliomas are characterized by their unique intratumoral and intertumoral heterogeneity, distinctive histological features of severe invasiveness, and most recently through genetic alterations such as copy number changes on chromosomes 7, 9, and 10 in adults, *IDH* mutations in older children and young adults, and histone mutations in diffuse midline gliomas that occur most commonly in children [3, 4, 5, 6, 7, 8, 9]. Gross total resection with adjuvant chemo/radiotherapy with temozolomide (TMZ) is the current standard of care in adults, although this approach is not applicable to diffuse midline gliomas (DMGs), which constitute most pediatric high‐grade gliomas. Whole‐genome, RNA, and epigenetic profiling has facilitated tumor categorization and provided strategies to incorporate molecularly targeted agents [10, 11]. However, the translation of large‐scale bioinformatic analysis into successful therapies will require leveraging actionable genetic modifications and combatting the frequent problem of tumor resistance, as well as the challenges posed by the blood–brain barrier (BBB).

In recent years, epigenetic modulators have shown some promise as potential targets for the treatment of high‐grade glioma. Panobinostat (Farydak®), also known as LBH589, an orally available FDA‐approved pan‐histone deacetylase inhibitor, has been used for treatment of various cancers [12] including gliomas [13, 14, 15, 16]. Panobinostat causes an increase in acetylation, leading to increased DNA transcription and translation of different proteins, cell cycle arrest, inhibition of cell proliferation, and induction of apoptosis. We [17] and others [18] have demonstrated its antitumor activity against pediatric, adult, and diffuse midline glioma *in vitro* and/or *in vivo*. This agent has logical applications in pediatric DMGs, which commonly have histone mutations [15, 19, 20] (NCT02717455) but has also shown preclinical activity in adult HGGs [17, 21].

Proteasome inhibitors are another agent class that has emerged as a potential targeted therapy for brain tumors. Proteasome inhibitors elevate cancer cell apoptosis by preventing the ubiquitin‐proteasome system from degrading damaged or misfolded proteins. Proteasome inhibitors promote cell death by altering protein homeostasis within the cell environment. Marizomib is a second‐generation, irreversible proteasome inhibitor (Pi) approved for treatment of multiple myeloma [22, 23] and tested in glioma [24]. Recent *in vitro* and *in vivo* studies demonstrated that marizomib significantly induces free radical production, inhibits cell proliferation, migration, and invasion and induces apoptosis in glioma. Furthermore, marizomib penetrates the BBB and elicits antitumor effects in orthotopic glioma models, suggesting the relevance of this agent for primary brain tumors [25].

We and others have shown that the combination of HDAC and proteasomal pathway inhibitors synergistically induces glioma cell death, suggesting the appeal of combination therapeutics to maximize treatment response [17, 26, 27, 28]. Based on these encouraging data, a phase 1 clinical study is being conducted to evaluate the safety, tolerability, and preliminary efficacy of the combination of marizomib and panobinostat in pediatric patients with DIPG (NCT04341311). Conversely, we have also shown that prolonged treatment with panobinostat‐ and bortezomib‐induced resistance, which was associated with upregulation of the NAD pathway [17], suggesting a role for dysregulation of energy metabolism as a therapy escape mechanism.

In this study, we build on these data and demonstrate that the combination of panobinostat and marizomib was very effective in pediatric DMG cell lines and also showed activity in adult non‐K27M‐mutant adult HGGs. However, similar to our previous observations with panobinostat and bortezomib [17], after a 60–120‐day treatment *in vitro*, cell populations resistant to the panobinostat‐marizomib combination emerged. RNA‐sequencing analysis showed a common pattern of activation of NAD pathway components and drivers of glycolysis in resistant vs. drug‐naïve cells, which led us to hypothesize that activation of energy metabolism provided a key mechanism of resistance. In agreement with that hypothesis, we observed that mitochondrial mass increased significantly in the resistant cells. Furthermore, ATP and NAD^+^ were produced at high rates, suggesting mitochondrial energetic pathways might be involved in resistant cell survival and growth. Because mitochondrial oxidative phosphorylation (OXPHOS) plays a critical role in modulating ATP supply, we hypothesized that pharmacological inhibitors of OXPHOS machinery (four enzyme complexes in the electron transport chain and a fifth enzyme complex that drives ATP synthesis) could cause functional disorders and cell death of the resistant cells. We examined the ability of 2‐deoxyglucose, a glucose analog, to inhibit hexokinase II and abolish ATP generation through the glycolytic pathway, as previously suggested [29], IACS‐010759, complex I inhibitor [30], currently in phase I clinical trial for solid neoplasms (NCT03291938), lonidamine, complex II and hexokinase II inhibitor [31, 32], extensively investigated in patients with breast and lung cancer [33, 34, 35], antimycin A, complex III inhibitor [36], FCCP, mitochondrial uncoupler, and Gboxin, ATP synthase inhibitor [37], in resistant cell lines. The clinical relevance of our *in vitro* findings was validated in an orthotopic mouse model harboring the H3.3‐K27M‐mutant DIPG‐013 cell line, which demonstrated independent activity of lonidamine in drug‐naïve cells, as well as dramatic potentiation of survival in PM‐resistant cells, indicating that targeting mitochondrial energetics has intriguing therapeutic potential in glioma.

## 2 Materials and methods

### 2.1 Reagents

Compounds utilized in our studies, including the small molecule inhibitors panobinostat, marizomib, 2‐DG, IACS‐010759, lonidamine, antimycin A, FCCP, and Gboxin, were purchased from Selleck Chemicals (Houston, TX, USA). Fluorescent probes were purchased from Invitrogen/Molecular Probes (Eugene, OR, USA) unless otherwise stated. The following antibodies were used: BiP (#3177), cleaved caspase 3 (#9664), cleaved caspase 7 (#8438), caspase 8 (#9746), Enolase‐2 (#24330), phospho‐H2AX (#80312), GAPDH (#2118), Hexokinase‐2 (#2867), PGC‐1α (#2178), RAD51 (#8875), and β‐Actin (#4970); all were from Cell Signaling Technology Inc., (Beverly, MA, USA).

### 2.2 Cell lines

Adult high‐grade glioma (aHGG) cell lines U87 (RRID:CVCL_4V16) and T98G (RRID:CVCL_0556) were obtained from the American Type Culture Collection (Manassas, VA, USA), and LNZ308 (RRID:CVCL_0394) was generously provided by Dr Nicolas de Tribolet (Division of Neurosurgery, University Hospital, Geneva, Switzerland). Of the pediatric high‐grade glioma (pHGG) cell lines, SJG2 (RRID:CVCL_M141) was a kind gift from Dr Chris Jones (Institute of Cancer Research, London, England), and KNS42 (RRID:CVCL_0378) was from the Japanese Collection of Research Bioresources Cell Bank. SF8628 (RRID:CVCL_IT46), a human DIPG cell line that harbors the histone H3.3 Lys 27‐to‐methionine (K27M) mutation, was purchased from Sigma‐Aldrich (St. Louis, MO, USA). All these cell lines were cultured in a growth medium composed of 10% fetal calf serum, l‐glutamine, 100 IU·mL^−1^ penicillin, and 100 mg·mL^−1^ streptomycin (Invitrogen, Carlsbad, CA, USA) supplemented with sodium pyruvate and nonessential amino acids. HSJD‐DIPG‐007 (RRID:CVCL_VU70) and HSJD‐DIPG‐013 (RRID:CVCL_C1MI), were gifts from Dr Angel Montero Carcaboso (Hospital Sant Joan de Déu, Barcelona), and were cultured in growth factor containing serum‐free media as described previously [38]. Cell line authentication was conducted by short tandem repeat analyses and cultures were tested for mycoplasma by the authors.

### 2.3 Flow cytometric assays

Flow cytometry was applied to measure cell viability, mitochondrial membrane potential, ROS generation, cell cycle profile, and mitochondrial mass.

For cell viability assay, fluorescent stain targeting different properties of apoptotic and nonapoptotic cells was performed using Annexin V‐FITC Conjugates for Apoptosis Detection kit (Thermo Fisher, Waltham, MA, USA, catalog number A13201) according to the manufacturer's protocol. After desired treatment conditions, cells (1.5 × 10^5^–2 × 10^5^) were washed with PBS, stained, and incubated for 10–15 min at room temperature as described previously [39].

To analyze mitochondrial membrane potential (Δψm), an important indicator of mitochondrial function and cell health, we used the carbocyanine dye DiOC6 (3,3′‐dihexyloxacarbocyanine iodide) as described previously [39]. Briefly, 2 × 10^5^ cells were seeded in complete growth media, allowed to attach overnight, and incubated with inhibitors or vehicles for the indicated duration. Then, cells were harvested, washed, and incubated for 30 min at 37 °C in 500 μL of 40 nm DiOC6, immediately followed by analysis on a flow cytometer with excitation and emission settings of 488 and 525 nm, respectively. Control experiments were performed in the presence of carbonyl cyanide m‐chlorophenyl hydrazone (CCCP), a specific inhibitor of mitochondrial membrane potential.

Biologically relevant ROS include hydrogen peroxide, superoxide anion, hydroxyl radicals, alkoxy radicals, and peroxy radicals and singlet oxygen. To analyze the inhibitor‐induced oxidative stress (the generation of ROS), we multiplexed two fluorescent dyes 2′,7′‐dichlorodihydrofluorescein diacetate (H2DCF‐DA) and hydroethidine (HE) to measure hydrogen peroxide and superoxide anion generation, respectively, as described elsewhere [40]. In brief, after overnight attachment in complete growth media, cells were incubated with inhibitors or vehicles for the indicated duration. Then, cells were harvested, washed, and incubated in 1 mL of PBS containing 2 μm of H2DCF‐DA and 1 μm of HE for 30 min at 37 °C in a dark environment. Flow cytometry was performed using a Becton Dickinson flow cytometer equipped with solid‐state blue laser, 488 nm, and emissions were detected through 520/20 and 575/25 nm filters, respectively.

The percentage of cells in G1 vs. S vs. G2/M (cell cycle profile) was determined as described previously [39]. In brief, after treatment with inhibitors or vehicle, cells were collected and fixed with 80% ethanol on ice for 30 min, then washed with PBS. Fluorescence of the PI‐stained cells was measured using a flow cytometer.

The fluorescent dye 10‐*N*‐nonyl‐acridine orange (NAO), a specific probe of cardiolipin (CL, a phospholipid found in the inner membrane of mitochondria) was used to measure mitochondrial mass per cell [41], as described previously [39]. NAO was excited by a broadband UV laser (488 ± 5 nm), and fluorescence emission was collected with a 525 ± 5 nm band‐pass filter.

Using Inkscape (The Inkscape Team), an Open Source vector graphics editor, the data were compiled into two‐dimensional histogram overlays for comparative analysis as described previously [39].

### 2.4 Cell viability analysis and the assessment of combination effects

Cells (1 × 10^3^ per well) were plated in 96‐well microtiter plates (Costar, Cambridge, MA, USA) in 75 μL of growth medium. On the following day, an equal volume of inhibitors (at 2× concentration) was added to each well using a multichannel pipette. Control cells received an equivalent amount of DMSO (vehicle). In parallel, replicates of 150 μL of media without cells served as the blanks. After 72 h of incubation at 37 °C, the number of viable cells was determined using a colorimetric cell proliferation assay kit (CellTiter96 Aqueous Non‐Radioactive Cell Proliferation Assay; Promega, Madison, WI, USA), as reported previously [17]. The degree of panobinostat and marizomib effect (synergistic or antagonistic or additive) was quantified using synergyfinder 2.0 (https://synergyfinder.fimm.fi), a stand‐alone web application for interactive analysis of drug combination [42, 43]. The data were analyzed using zero interaction potency (ZIP) model, which offers an increased power (compared with other existing reference models such as highest single agent model, Loewe additivity model, and Bliss independence model) to differentiate between various classes of drug combinations, and may therefore provide an improved means for understanding their mechanisms of action toward clinical translation [44].

### 2.5 Clonogenic growth assay

The ability of a single cell to survive and grow into a colony after inhibitor(s) treatment was also assessed using a clonogenic assay as described previously [27].

### 2.6 Generation of PM‐resistant cell lines

Resistant cell lines used in this study were derived from each original parental cell line by continuous exposure to the inhibitors. Adult HGG, pediatric HGG, and pediatric DIPG cells were treated with the combination of both starting with a dose that was approximately 10% of the clinically relevant concentration (the median maximum concentration of panobinostat was 21.2 ng·mL^−1^ [45] and 2.8 to 57.8 ng·mL^−1^ for marizomib [23]). Control cells received an equal amount of vehicle (DMSO). Viable cells were isolated using a BD ARIAII cell sorter (BD Biosciences, San Jose, CA, USA), washed with PBS, and cultured in growth media containing double the concentration of inhibitors. This process was repeated until the cells grew at a comparable rate in the presence of panobinostat and marizomib (25 nm each). The resistance‐development period was about 2–4 months. During this time, cell viability, cell cycle profile, and colony‐forming ability were assessed between drug‐naïve control and resistant cells to determine changes in sensitivity to the inhibitors.

### 2.7 Quantitative real‐time PCR analysis, RNA sequencing, gene enrichment, and pathway analysis

Real‐time PCR analysis was performed to measure mRNA expression levels in drug‐naïve vs. inhibitor‐treated cells. Total RNA from four biological replicates was isolated using a Qiagen RNeasy Mini kit (Germantown, MD, USA) according to the manufacturers' instructions and quantified by measuring the optical density (NanoDrop ND‐1000 Spectrophotometer, NanoDrop Technologies, Menlo Park, CA, USA). Using Applied Biosystems (Foster City, CA, USA) reverse transcription assay kit, 1 μg of total RNA was converted to first‐strand cDNA in a volume of 20 μL containing RNA template, 100 mm gene‐specific primers, reverse transcription buffer, 5 mm MgCl_2_, 10 mm DTT, RNase inhibitor, and reverse transcriptase. The first strand of cDNA was stored at −20 °C. We performed one‐step quantitative real‐time PCR using Reverse Transcriptase with Power SYBR Green (both from Applied Biosystems) on the StepOnePlus Instrument (Applied Biosystems) in 96‐well microtiter plates. Amplification protocols were followed with an initial denaturation step at 95 °C for 5 min followed by 40 cycles of 95 °C for 15 s, 60 °C for 30 s, and 72 °C for 30 s in 25 μL reaction volume with 10 ng of cDNA template. The change in gene expression [(ΔΔ*C*_T_ where *C*_T,Target_ − *C*_T,ẞ‐actin_)_Time *x*_ − (*C*_T,Target_ − *C*_T,ẞ‐actin_)_Time 0_] normalized to a housekeeping gene (ẞ‐actin) was assessed as described previously [46, 47]. The primer sets [PGC‐1, CCTGTGGATGAAGACGGATT (forward primer), TAGCTGAGTGTTGGCTGGTG (reverse primer) (IDT reference number, 409437753); Actin, TGTCCACCTTCCAGCAGATGT (forward primer), AGCTCAGTAACAGTCCGCCTAGA (reverse primer), (IDT reference number, 409437765)] used in this study were purchased from Integrated DNA Technologies (IDT, Coralville, IA, USA).

After RNA extractions (as described above), strand‐specific RNA‐Seq library preparation and subsequent sequencing were performed at Novogene Biotech (Beijing, China) using standard Illumina protocols carried out on the Illumina NovaSeq 6000 platform as per the manufacturer's instructions (Novagene Genomic Services (Durham, NC, USA)) as described before [48]. Short read sequences (150 bp, mate paired) obtained from sequencing were trimmed and further mapped to the reference human genome (hg19). Paired gene sequences were aligned using the bowtie (v2.3.5.1) algorithm employed in CLC Genomics Workbench 2020 ((Ensemble version 54), Qiagen, CLC Workbench 2020) [49]. Genes with Log_2_(Transcript per million (TPM) + 1) values < 2 were considered to not be expressed at a sufficient level to permit reliable quantification and were removed. Sample counts were then normalized to counts per million (CPM) and transformed (Log2CPM + 2) using edge r package in the r computing environment. iDEP.94 DESeq2 statistical packages in r were used to identify significant changes in gene expression between experimental conditions [50, 51]. Significance was defined as genes having an absolute fold change > 1.5 and a false discovery rate (FDR) < 0.05. Significant gene lists were analyzed using the enrichr Package in python [52, 53, 54]. Differentially expressed genes were also analyzed using Gene Set Enrichment Analysis (GSEA) utilizing Genematrix h.all.v7.5.symbols.gmt [Hallmarks] [55, 56].

### 2.8 Adenosine triphosphate (ATP) assay

The amount of ATP was measured by using an ATP Colorimetric/Fluorometric Assay Kit, according to the manufacturer's protocol (BioVision, catalog number K354, Waltham, MA, USA). The cells were seeded onto 6‐well plates at 2.5 × 10^5^ cells per well and allowed to attach overnight. After treatment with an inhibitor or vehicle for the desired duration, cells were collected, and treated with lysis buffer from the ATP detection kit. The lysate was centrifuged at 12 000 × ***g*** for 10 min at 4 °C, and then, the supernatant was moved to a fresh tube. The protein concentration was determined using a Protein Assay Reagent (Pierce Chemical, Rockford, IL, USA) using BSA as standard. An equal amount of protein was taken; subsequently, the lysate was incubated with enzyme mix and the developer solution (provided in the kit) in 96‐well plates in triplicate. The plate was incubated at room temperature for 30 min in the dark and absorbance was measured at 450 nm by BioTek, Synergy HTX multimode plate reader (Winooski, VT, USA).

### 2.9 NAD ^+^ quantification

Total intracellular NAD^+^ content was measured as described previously using the colorimetric assay kit (BioVision, Milpitas, CA, USA) [17].

### 2.10 Liquid chromatography–high‐resolution mass spectrometry (LC‐HRMS) analysis of TCA cycle metabolites

Drug‐naïve or PM‐resistant cells were collected for steady‐state metabolomics analysis as described before [57]. In brief, cells were pelleted at 2 × 10^6^ cells per replicate, completed in triplicate. Pellets were flash frozen, and metabolite extraction was completed using ice‐cold 80 : 20 methanol:water with 0.1% formic acid that included internal standards, creatinine‐d3, alanine‐d3, taurine‐d4 and lactate‐d3 at a final concentration of 10 μm. After 3 min of vortexing, the supernatant was cleared of protein by centrifugation at 16 000 × ***g***. Samples of cleared supernatant (2 μL) were injected via a Thermo Vanquish UHPLC and separated over a reversed‐phase Thermo HyperCarb porous graphite column (2.1 × 100 mm, 3 μm particle size) maintained at 55 °C. For the 20‐min LC gradient, the mobile phase consisted of the following: solvent A (water/0.1% FA) and solvent B (ACN/0.1% FA). The gradient was the following: 0–1 min 1%B, with an increase to 15%B over 5 min, increasing to 98%B over 5 min, holding at 98%B for 5 min, and then equilibration at 1%B for 5 min. The Thermo ID‐X tribrid mass spectrometer was operated in both positive and negative ion mode, scanning in ddMS2 mode (2 μscans) from 70 to 800 *m/z* at 120 000 resolution with an AGC target of 2e5 for full scan and 2e4 for MS2 scans using HCD fragmentation at stepped 15, 35, 50 collision energies. Source ionization settings were 3.0 and 2.4 kV spray voltage, respectively, for positive and negative modes. Source gas parameters were 35 sheath gas, 12 auxiliary gas at 320 °C, and 8 sweep gas. Calibration was performed prior to analysis using the PierceTM FlexMix Ion Calibration Solutions (Thermo Fisher Scientific). Integrated peak areas were then extracted manually using quan Browser (Thermo Fisher xcalibur ver. 2.7).

### 2.11 Pyruvate dehydrogenase activity assay

Pyruvate dehydrogenase (PDH), a mitochondrial multienzyme, that catalyzes the conversion of pyruvate to acetyl‐coenzyme A was assessed using the Pyruvate Dehydrogenase Activity Colorimetric Assay Kit from BioVision (Milpitas, CA, USA; Catalog number K679‐100) as described elsewhere [39]. Triplicate assays were performed on each sample with appropriate positive and negative controls provided by the manufacturer. The plate was incubated at 37 °C in the dark and absorbance was measured using an HTX Multimode Plate Reader (BioTek Instruments Inc., Model number S1FA; Winooski, VT, USA) at 450 nm for a 30‐min interval. An NADH standard curve was prepared in parallel, and the slopes of the kinetic measurements were used to calculate the rate of NADH produced per minute per microgram (PDH activity) of protein as described in the protocol.

### 2.12 LC–MS‐based quantitative measurement of NAD ^+^ metabolites

Quantitative NAD^+^ metabolome, as defined here, includes dinucleotides, nucleotides, nucleosides, and nucleobases that were measured as described previously [58]. In brief, drug‐naïve or PM‐resistant cells were counted, pelleted, and washed once in ice‐cold potassium‐buffered saline. Then, cells were resuspended in 300 μL of a 75% ethanol/25% 10 mm HEPES, pH 7.1 v/v (buffered ethanol) solution, preheated to 80 °C. Samples were shaken at 100 × ***g*** in an 80 °C block for 3 min. Soluble metabolites were separated from particulate by refrigerated microcentrifugation (10 min, 16 000 × ***g***). Though the ethanol‐soluble extract contains all the metabolites of interest, the weight of the particulate can be used to determine the optimized resuspension volume for dried metabolites. Then, both the particulate and soluble metabolites were dried by speed vacuum at 40 °C. For LC–MS analysis, the pellet was resuspended and diluted two‐fold into two different metabolite standards, and 2.5 μL of the resulting material was injected and analyzed. Using internal standards, intracellular metabolites per million cells were reported on a pmol scale.

### 2.13 Hexokinase enzymatic activity assays

Hexokinase (HK) enzymatic activities were assessed by the colorimetric method described by the manufacturer (Catalog Number MAK091, Sigma). After adding the reaction mix to the samples, this assay was performed in a 96‐well plate at room temperature at 450 nm.

### 2.14 Animal studies

All animal procedures were carried out ethically according to protocols approved by the institutional animal care and use committee (IACUC) at the University of Pittsburgh (approval number 20108110, PI: Agnihotri). The *in vivo* model was generated using 4‐week‐old NOD‐SCID female mice (NOD.Cg‐*Prkdc* ^scid^ /J Strain, catalog number 001303, Jaxson Labs, Bar Harbor, ME, USA) injected with DIPG‐013 drug‐naïve or DIPG‐013 PM‐resistant cells. Cells (2.5 × 10^4^) were resuspended in 2 μL of PBS and injected into the pons/midbrain via stereotactic frame (Stoelting, Wood Dale, IL, USA) and automated cell injector (Stoelting) over a 4‐min period. Coordinates were as follows from the lambda suture (*x* = 1 mm, *y* = 0.8 mm, *z* = −3.5 mm). Mice were injected and separated into two groups: control and experimental. Experimental mice were treated with two rounds of five doses of lonidamine (50 mg·kg^−1^), for a total of 10 doses. Each round consisted of 1 dose per day, with 2 days of no drug in between rounds. Control mice were dosed with vehicle (5% DMSO in Tris/glycine buffer). Mice were kept in a room at an ambient temperature (73–74°F), with 30% humidity, a dark–light cycle of 14–10 h, in a mouse satellite box with no restrictions on food or water. Mice were monitored for any signs of decline in health daily. Animals losing ≥ 10% body weight or having other symptoms such as hydrocephalus, or neurological duress were sacrificed humanely as defined by IACUC. Survival distribution was determined using Kaplan–Meier analysis [48].

### 2.15 Statistical analysis

All experiments were performed in biological triplicates unless otherwise stated. The data were expressed as mean ± SD. Statistical analyses were conducted using prism 6.0 graphpad software (GraphPad Software, Boston, MA, USA). To identify differences among subgroups, ANOVA analysis was conducted for multigroup comparisons followed by the *post‐hoc* Tukey's test. Direct comparisons were conducted using an unpaired two‐tailed Student *t*‐test.

## 3 Results

### 3.1 Cotreatment with marizomib and panobinostat promotes apoptosis in pediatric and adult glioma cell lines

**Fig. 1:**
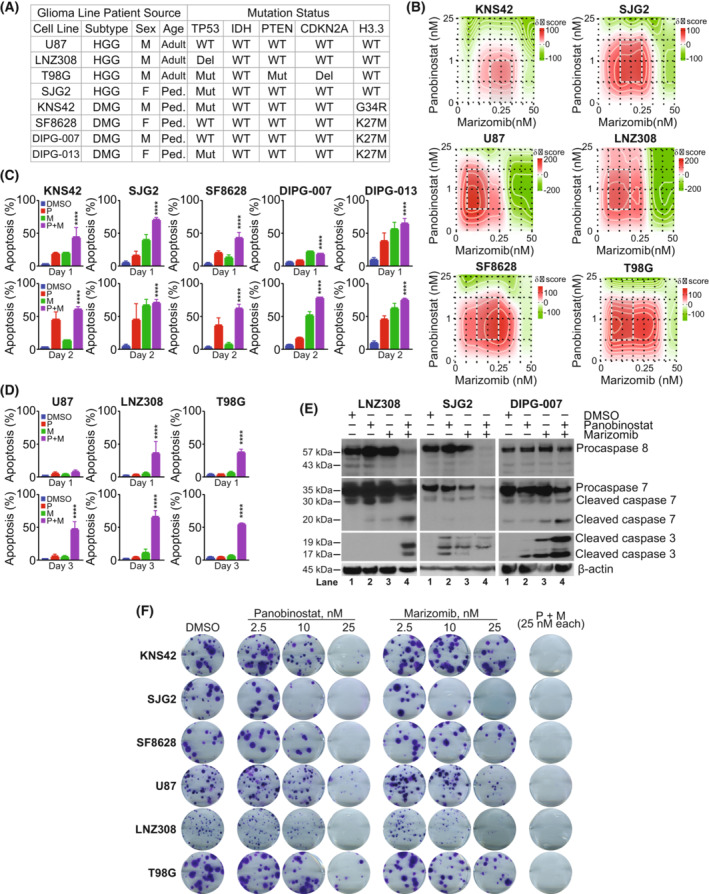
Cotreatment of panobinostat and marizomib promotes apoptosis in pediatric and adult glioma cell lines. (A) Genetic and biological characterization of the established glioma cell lines used in this study (HGG, high‐grade glioma; DMG, diffuse midline glioma; M, male; F, female; Ped, pediatric; WT, wild‐type; Del, deletion; Mut, mutation). The TP53 gene at chromosome 17p13.1, plays a critical role in the cell cycle, cellular responses to DNA damage, cell death, and differentiation. Isocitrate dehydrogenase 1 and 2 (IDH1 and IDH2) enzymes convert isocitrate to α‐ketoglutarate (α‐KG) through oxidative decarboxylation. IDH1/2 mutations introduce a gain‐of‐function activity to the enzyme that leads to accumulation of (+)‐2‐hydroxyglutarate (2‐HG). Phosphatase and TENsin homolog (PTEN), a gene located in the 10q23 region of chromosome 10 encoding for a 403‐aminoacid multifunctional protein, a crucial tumor suppressor, exhibits phosphatase‐dependent PI3K‐AKT–mTOR pathway activities to maintain cellular homeostasis. Cyclin Dependent Kinase Inhibitor 2A (CDKN2A), a tumor suppressor gene located at chromosome 9 blocks the progression of cell cycle from G1 to S phase. Recurrent mutations in H3 histone, family 3A (H3F3A), which encodes the replication‐independent histone 3 variant H3.3 led to amino acid substitutions at two critical positions within the histone tail (K27M, lysine at position 27‐to‐methionine; G34R, glycine at position 34‐to arginine; G34V, glycine at position 34‐to valine) involved in key regulatory post‐translational modifications. H3F3A mutations are highly prevalent in children and young adults. Histone 3 lysine 27‐to‐methionine (H3‐K27M) mutations most frequently occur in diffuse midline gliomas (DMGs). (B) Pediatric high‐grade glioma (KNS42, SJG2) or adult high‐grade glioma (U87, LNZ308, and T98G) and pediatric brain stem glioma (SF8628) were seeded in 96‐well plates in 75 μL of complete media. On the following day, cells were treated with an equal volume of panobinostat (0, 0.25, 1, 5, and 25 nm) or marizomib (0, 0.01, 0.05, 0.25, 1, 5, 25, and 50 nm) and their combination on cell proliferation in six glioma cell lines (KNS42, SJG2, U87, LNZ308, SF8628, and T98G). After 72 h of incubation at 37 °C, the number of viable cells was determined using a colorimetric cell proliferation assay kit as described in the Section 2. The degree of panobinostat and marizomib effect (synergistic or antagonistic or additive) was quantified using synergyfinder 2 (https://synergyfinder.fimm.fi), a stand‐alone web application for interactive analysis of drug combination. The data (*n* = 3) were analyzed using zero interaction potency (ZIP) model, which offers an increased power to differentiate between various classes of drug combinations for understanding their mechanisms of action toward clinical translation. The representative interaction landscape (heatmap) from three separate experiments for six different cell lines indicates that the combination of panobinostat and marizomib was able to achieve a higher effect (red‐shaded area) than the single agent. (C, D) KNS42, SJG2, SF8628, DIPG‐007, and DIPG‐013 (C); U87, LNZ308, and T98G cells (D) (1 × 10^5^ per well) were plated in 6‐well microtiter plates. On the following day, cells were treated with panobinostat (25 nm, P), marizomib (25 nm, M), or both (P + M, 25 nm each) in combination. Control cells received an equivalent amount of DMSO (vehicle). After the indicated duration, the cells were stained with Annexin V and propidium iodide, and cell viability was assessed using flow cytometry as described in the Section 2. Values are represented as mean ± standard deviation of three separate experiments. Results were analyzed by Tukey's ANOVA (C, D, ****P < 0.0001, vehicle‐treated cells vs. combination of panobinostat and marizomib). (E) Logarithmically growing adult high‐grade glioma cells (LNZ308) were treated with panobinostat (25 nm) or marizomib (25 nm) or the combination of both (Pano + Mari, 25 nm each) for 72 h (left panel) and pediatric cells (SJG2 and DIPG‐007) for 48 h (middle and right panel). Twenty micrograms of protein were loaded on a sodium dodecyl sulfate‐polyacrylamide gel and probed with the indicated antibodies by western blotting. These experiments were performed at least three times, and a representative blot is presented. (F) Pediatric (KNS42, SJG2, and SF8628) and adult (U87, LNZ308, and T98G) cells were treated with indicated concentrations of panobinostat (2.5, 10, and 25 nm) or marizomib (2.5, 10 and 25 nm) or the combination of both (P + M, 25 nm each) for 24 h. Control cells received vehicle (DMSO). Clonogenic assay was performed as described in the Section 2. These experiments were performed at least three times, and a representative image is presented.

Our previous studies have shown that combination of HDAC and proteasome inhibitors provided inhibition of glioma growth *in vitro*, and dramatic potentiation of cell killing that far exceeded the effects of either agent alone, although these studies incorporated non‐brain‐penetrant agents [17, 27]. Because genetic heterogeneity at the cellular level provides multiple mechanisms for therapeutic resistance in pediatric and adult gliomas [59], we selected a panel of glioma cell lines to test the hypothesis that panobinostat or marizomib, two agents with evidence of BBB penetration, or their combination could alter glioma viability regardless of genetic status (Fig. 1A). We investigated the effect of panobinostat (0, 0.25, 1, 5, and 25 nm) and marizomib (0, 0.01, 0.05, 0.25, 1, 5, 25, and 50 nm) and their combination on cell proliferation in six glioma cell lines (KNS42, SJG2, SF8628, U87, LNZ308, and T98G). We used synergyfinder 2.0 [43], a web application that enabled us to analyze the effect as a single agent and the combinations in an interactive manner for an unbiased analysis. Using a zero interaction potency (ZIP) reference model [44], the dose–response matrix and the degree of a drug combination effect were analyzed (Table S1). Although the pattern of synergy varied as a function of concentration (Table S1), the interaction landscape (Fig. 1B) indicated that the combination of panobinostat and marizomib achieved a synergistic effect in all six cell lines regardless of genetic status. To ascertain whether the observation was due to cytostatic or cytotoxic effects, KNS42, SJG2, SF8628, DIPG‐007, and DIPG‐013 cells were exposed to either panobinostat (25 mm), marizomib (25 nm) as a single agent or the combination for the indicated duration, and cell death was assessed by Annexin V and propidium iodide (PI) staining by flow cytometry. Although there was a minimal effect of panobinostat and marizomib after 1 day of treatment (~ 20% cell death in KNS42, SJG2, and SF8628), the combination of the two agents produced ~ 75%, 90%, and 50% of cell death in KNS42, SJG2, and SF8628, respectively (Fig. 1C). While the combination of these two inhibitors produced only 25% of cell death in DIPG‐007 cells (after 1‐day exposure, ****P < 0.0001 vehicle‐treated cells vs. panobinostat and marizomib cotreated cells, Fig. 1C upper panel), cotreatment with these agents for 2 days caused ~ 90% cell death. When we examined these combinations to extend their applicability in adult GBM patients, unlike pediatric glioma cells, flow cytometric analysis showed that panobinostat or marizomib alone induced only few PI‐positive cells (after 3 days of exposure), but the combination significantly induced cell death in a time‐dependent fashion, suggesting that combination of two agents may be necessary to induce cell death in adult high‐grade glioma cell lines (Fig. 1D). We also examined the maturation of caspases, which play key roles that culminate in programmed cell death (apoptosis). As shown in Fig. 1E, after a longer incubation period (aHGG cell line LNZ308, 72 h), we noticed a significant reduction in procaspase‐8 (lane 4), and increased appearance of active caspase 7, 3 (in lane 4 but not in lane 1–3), a very similar trend seen by the annexin assays (i.e., decrease in the viable cells after cotreatment but not to the single agent alone). However, like cell viability assays, exposure to panobinostat or marizomib (as a single agent for 48 h) or the combination resulted in an increased maturation of caspases (SJG2 and DIPG‐007, Fig. 1E), suggesting that pediatric glioma cells are more sensitive to panobinostat and marizomib than adult glioma cell lines. However, it should also be noted that regardless of genetic status, at physiologically relevant concentrations, the combination of panobinostat and marizomib resulted in a synergistic inhibition of cell growth and induction of apoptosis.

To assess whether every cell in the population could re‐enter the cell cycle and grow into a colony (to determine the long‐term effectiveness of the inhibitors), we performed clonogenic growth assays, an *in vitro* cell survival assay. Six different glioma cells were incubated with either as a single agent (multiple concentrations) or the combination for 24 h. After 1 day, the inhibitor was removed and then cells were cultured in an inhibitor‐free medium for 14 additional days. As shown in Fig. 1F, although there is a significant inhibition of colony size between vehicle‐treated vs. single agent‐treated cells, only a fraction (or none) of the seeded cells treated with the combination of panobinostat and marizomib retains the capacity to produce colonies. Together, these assays yielded information about differences in sensitivity to the inhibitors and implied that combining panobinostat and marizomib may be beneficial for the long‐term treatment outcomes of both pediatric and adult glioma patients.

### 3.2 Cotreatment of panobinostat and marizomib induces mitochondrial dysfunction

**Fig. 2:**
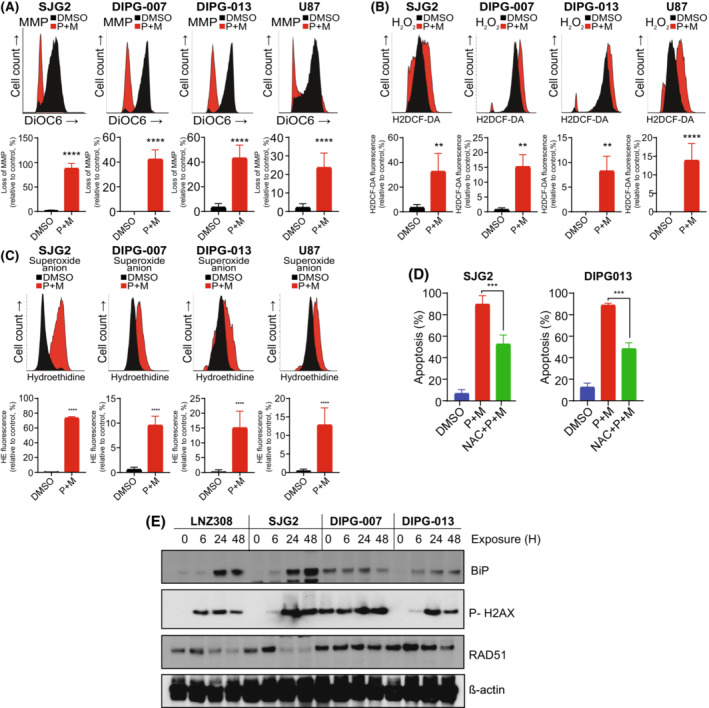
Cotreatment of panobinostat and marizomib induces mitochondrial dysfunction. (A–C) Pediatric and adult glioma cell lines were treated with the combination of panobinostat and marizomib (Pano + Mari, 25 nm each) or vehicle (DMSO) for 24 h. Then, the cells were labeled with DiOC6 to measure mitochondrial membrane potential (A). A representative FACS plot and the data obtained from three separate experiments demonstrated a loss of mitochondrial membrane potential (A, upper panel). The loss of mitochondrial membrane potential (left‐shifted population) was quantified. The values are represented as mean ± standard deviation of three separate experiments (****P < 0.0001; vehicle‐treated control vs. inhibitor‐treated cells; unpaired two‐tailed *t*‐test; A, lower panel). The cells were labeled with H2DCF‐DA and hydroethidine (HE) to analyze hydrogen peroxide (B) and superoxide anion (C), respectively, by flow cytometry. All experiments were performed at least three times. A representative FACS histogram is shown in the upper panel. Treatment with inhibitor was accompanied by an increase of H2DCF‐DA fluorescence (shift to the right; B, upper panel) and HE fluorescence (shift to the right; C, upper panel), which is proportional to the intracellular H_2_O_2_ and superoxide anion, respectively. Quantitative data from three separate experiments are presented in the lower panel (values are represented as mean ± standard deviation: B, H_2_O_2_ content; unpaired two‐tailed *t*‐test **P < 0.005, ****P < 0.0001 and C, superoxide anion; ****P < 0.0001; unpaired two‐tailed *t*‐test). (D) Cells were treated with panobinostat and marizomib in the presence or absence of NAC (0.25 mm), reactive oxygen species scavenger. After 24 h, cells were stained with annexin V and propidium iodide (PI), and quantitative measurements of apoptosis were performed by flow cytometry. Graph represents the percentages of apoptotic cells acquired from three independent experiments for each cell type (mean ± SD). Statistical significance was assessed with Tukey's ANOVA (***P < 0.0001). (E) After treatment with the combination of panobinostat and marizomib (25 nm each) for the indicated duration, whole cell extracts were subjected to western blot analysis. Equal amounts of protein were separated by SDS/PAGE and subjected to western blot analysis with the indicated antibodies. β‐actin served as a loading control. These experiments were performed at least three times, and a representative image is presented.

Because loss of mitochondrial membrane potential is believed to be a key upstream event during apoptosis, and maintenance of a proper mitochondrial membrane potential (Δψm), a critical bioenergetic parameter, is essential for oxidative phosphorylation, ATP synthesis, and cell survival, we used the cationic dye, DiOC6 [60] to measure the mitochondrial membrane potential. As depicted in Fig. 2A, cells treated with the combination of panobinostat and marizomib resulted in the loss of mitochondrial membrane potential. Cells exposed to panobinostat and marizomib also exhibited a significant increase in both H_2_O_2_ (Fig. 2B) and superoxide anion (Fig. 2C). Pretreating cells with NAC, free radical scavenger, significantly protected cells from undergoing apoptosis (Fig. 2D), suggesting that the increase in ROS can cause cell death in panobinostat and marizomib treated glioma cells. We also examined the expression level of BiP, a widely used marker for ER stress [61], phosphorylation of histone H2AX, a marker that correlates well with DNA damage [62] and Rad51, a key molecule associated with the regulators of DNA fidelity through diverse roles in double‐strand break and repair [63]. Cotreatment of panobinostat and marizomib significantly increased ER stress, and loss of repair pathways to cope with DNA damage from the inhibitors (Fig. 2E). Together, these results demonstrate the importance of HDAC and proteasome signaling pathway for GBM cell growth and that the lack of it hinders proliferation while facilitating the entry of cells into apoptosis.

### 3.3 Enhanced glycolysis and the mitochondrial TCA cycle metabolites are associated with panobinostat and marizomib‐induced resistance

**Fig. 3:**
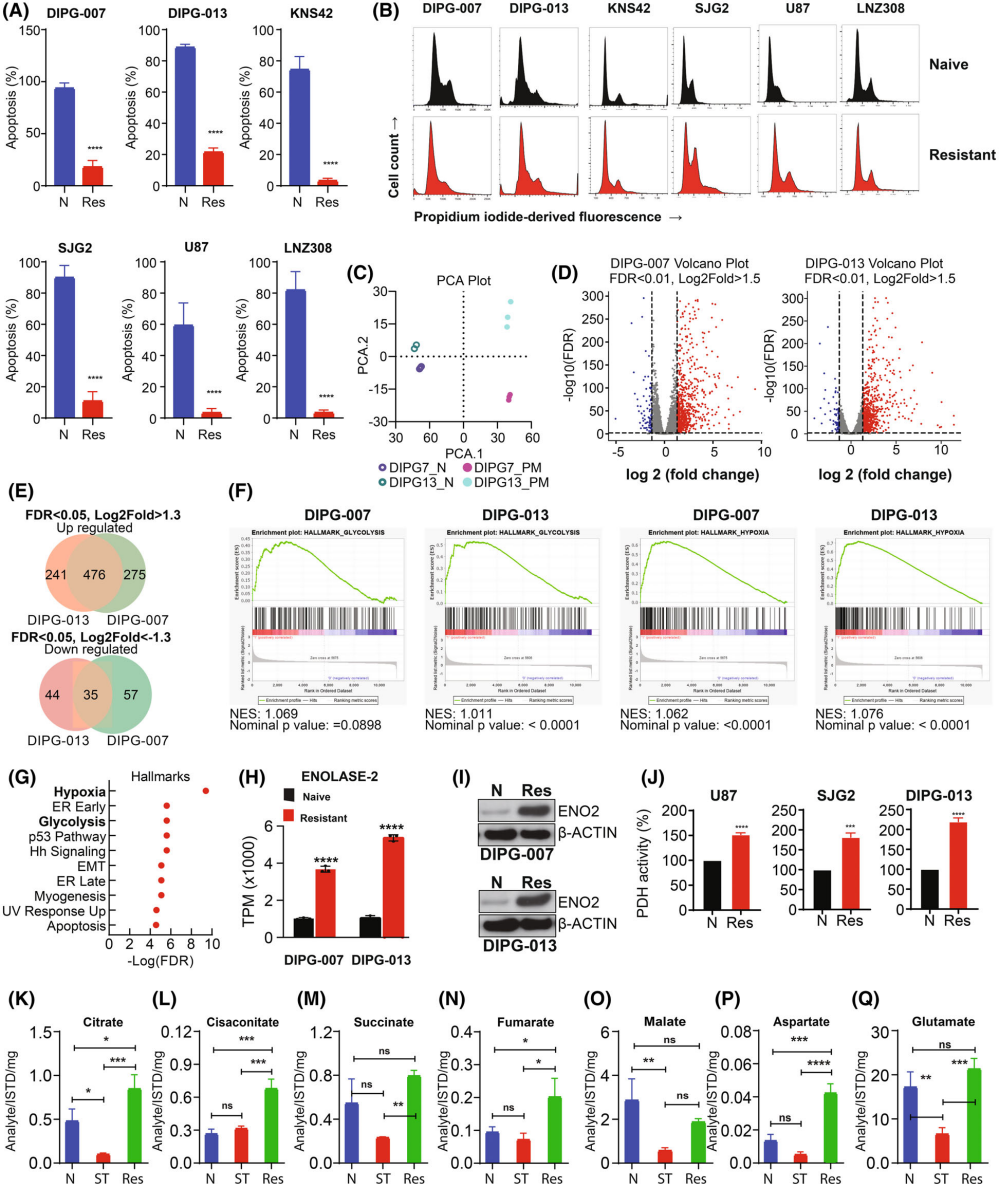
Resistance is associated with an enhanced glycolytic phenotype. (A) Drug‐naïve (N) or PM‐resistant (Res) cells were treated with the combination of panobinostat and marizomib (25 nm each) for 48 h. After harvesting, cells were labeled with annexin V and propidium iodide, and cell viability was assessed by flow cytometry. The results represent the mean of three independent experiments. Error bars indicate ± SD (****P < 0.0001; naive‐treated vs. resistant cells; unpaired two‐tailed *t*‐test). (B) Drug‐naïve (upper panel) or PM‐resistant cells (lower panel) were allowed to attach overnight in a 6‐well plate. Cells were harvested and stained with propidium iodide (PI). Cell cycle profile was assessed by flow cytometry. Representative images acquired from three independent experiments for each cell type are shown. (C) Principal component analysis (PCA) plot of RNA‐seq data comparing drug‐naïve control DIPG‐0007 and DIPG‐013 with PM‐resistant cell lines (*n* = 3). (D) Volcano plot of differential gene expression (FDR, false discovery rate) analysis of RNA‐seq data comparing drug‐naïve vs. PM‐resistant DIPG‐007 (left panel) and DIPG‐013 (right panel) cell lines (*n* = 3). The number of upregulated genes that are highlighted in red FDR < 0.01, Log2fold > 1.5, and downregulated genes that are highlighted in Blue FDR < 0.01, Log2fold < −1.5 (*n* = 3). (E) Venn‐diagram showing the number of upregulated genes (upper panel) and downregulated (lower panel) genes (*n* = 3). (F) Gene set enrichment analysis (GSEA) plots (NES, normalized enrichment score) showing glycolysis and hypoxia gene sets of DIPG‐007 and DIPG‐013 of drug‐naïve vs. PM‐resistant cell lines (*n* = 3). (G) Hallmark analysis of drug‐naïve vs. PM‐resistant DIPG‐013 cell lines reveals enrichment of hypoxia and glycolytic signatures (among top 10; *n* = 3). (H) Transcripts per million (TPM) of enolase‐2 in DIPG‐007‐ and DIPG‐013‐naïve vs. ‐resistant cell lines, the results were generated from three biological replicates (****P < 0.0001 naïve vs. resistant, analysis was performed as one‐way ANOVA). Data are displayed as mean with standard deviation. (I) Drug‐naïve (N) or PM‐resistant cells (Res) were allowed to attach overnight in a 6‐well plate. Whole cell lysates were prepared, and western blot analysis was performed with the indicated antibodies (ENO2, enolase‐2). Representative blots acquired from three independent experiments for each cell type are shown. (J) Drug‐naïve (N) and PM‐resistant (Res) U87, SJG2, and DIPG‐013 cells were seeded in 6‐well plates. On the following day, cellular pyruvate dehydrogenase (PDH) activity was assessed as described in the Section 2. The results represent the mean ± SD of three independent experiments (***P < 0.0005; ****P < 0.0001; unpaired two‐tailed *t*‐test). (K–Q) Quantification of TCA cycle‐related metabolites from U87‐resistant cells and drug‐naïve cells treated with the combination of panobinostat and marizomib for 72 h (ST, short‐term). Untreated control cells (N) received vehicle (DMSO). Citrate (K), cis‐aconitate (L), succinate (M), fumarate (N), malate (O), aspartate (P) and glutamine (Q). The values are represented as mean ± standard deviation of three separate experiments (K–Q). Statistical significance was assessed with Tukey's ANOVA (ns, nonsignificant, *P < 0.05, **P < 0.005, ***P < 0.0005, and ****P < 0.0001).

Although the combination of panobinostat and marizomib holds therapeutic promise, intrinsic or acquired resistance continues to be a major limiting factor for treatment response [64]. To examine the mechanism of panobinostat‐marizomib resistance and define a strategy to overcome resistance, we chose a panel of glioma cells, representing both pediatric and adult, which harbor significant genetic heterogeneity that was exposed to physiologically relevant drug concentrations for several weeks, leading to the selection of a PM‐resistant population (Fig. S1A). Cell viability assay indicated that cotreatment with panobinostat and marizomib significantly decreased cell survival of drug‐naïve cells at concentrations that were well tolerated by resistant cells (Fig. 3A). We noticed no discernable change in cell cycle profile between drug‐naïve vs. resistant cells (Fig. 3B) or the colony‐forming ability (Fig. S1B). Of note, while the mechanism is yet to be determined, we observed a notable time difference to generate resistance cell populations in the pediatric cell lines compared with the adult counterparts (Fig. S1C).

To identify genes and gene expression patterns that may contribute to resistance, we performed RNA sequencing with biological triplicates of drug‐naïve and PM‐resistant cells (Table S2). Principal component analysis (PCA) was used for multidimensional scaling to plot RNA‐sequencing data of DIPG‐007‐ and DIPG‐013‐naïve vs. ‐resistant cells and determined that each cell line clustered independently from one another (Fig. 3C). Significant differentially expressed genes were identified with an FDR < 0.05 and an absolute Log_2_(Fold Change) > 1.5 (Fig. 3D). Strikingly, there were 476 significantly upregulated genes (48%) common among both DIPG‐007 and DIPG‐013 datasets (Fig. 3E). Pathway analysis using significantly upregulated genes across both datasets identified several known and novel pathways enriched in resistant cells. Of interest, glycolysis and hypoxia‐related gene expression (Fig. 3F) were significantly enriched in resistant cells compared with naïve controls (Fig. 3G). We focused on glycolysis as the majority of genes in the hypoxia signature were glycolytic in nature (X/Y) and our experiments were conducted in normoxia with glycolytic intermediates known to elicit hypoxic/pseudohypoxic environments [65, 66, 67, 68]. Results from our bioinformatic study (Fig. 3H) and western blot analysis (Fig. 3I) support a correlation between resistance and enolase‐2 expression, an enzyme involved in the penultimate step of glycolysis that converts 2‐phosphoglycerate to phosphoenolpyruvate (Fig. S2A), and functions as a crucial activator of other oncogenic pathways, leading to cell proliferation and treatment resistance [69, 70, 71].

After glucose is metabolized to pyruvate, the enzyme pyruvate dehydrogenase (PDH) regulates the oxidation of pyruvate to acetyl‐coenzyme A (acetyl‐CoA; Fig. S2A). As shown in Fig. 3J, compared with drug‐naïve controls, PDH activity was rapidly increased in the resistant cells. Then, we investigated TCA cycle metabolites (Fig. S2A), which play a pivotal role in cell metabolism and drug resistance [72]. We observed a significant increase in the TCA metabolites such as citrate (Fig. 3K), cis‐aconitate (Fig. 3L), succinate (Fig. 3M), fumarate (Fig. 3N), and malate (Fig. 3O) in the resistant cells compared with untreated cells or cells treated for 72 h. The nonessential amino acid aspartate increased substantially (~ 3‐fold) in PM‐resistant cells (Fig. 3P), suggesting that a change in aspartate levels might play an important role in the resistant phenotype [73, 74]. Similarly, glutamate (Fig. 3Q), another nonessential amino acid, which plays a crucial role in cell metabolism by participating in the TCA cycle, nucleotide biosynthesis, and generation of glutathione necessary for redox balance and cellular homeostasis [75], substantially increased in PM‐resistant cells.

Because glutamine and other TCA metabolites contribute directly to *de novo* biosynthesis of both purines and pyrimidines [76], which are essential to maintain cellular homeostasis and growth, we then ascertained the levels of cytidine (Fig. S2B), a substrate for the salvage pathway of pyrimidine nucleotide synthesis, inosine (Fig. S2C), a natural purine nucleoside (a product of adenosine), and inosine 5′‐monophosphate (IMP; Fig. S2D), an intermediate in *de novo* purine biosynthesis that can be converted to either GMP or AMP, suggesting that the resistant cells use various pathways to meet their purine and pyrimidine nucleotide requirements.

### 3.4 Increased mitochondrial mass, ATP, and NAD ^+^ content in the resistant cells

**Fig. 4:**
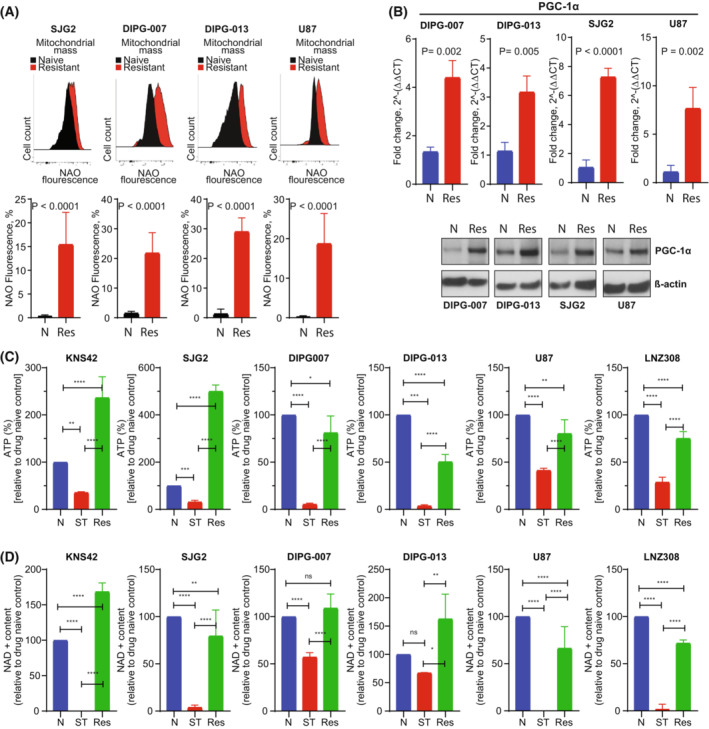
Mitochondrial mass, NAD, and ATP were increased in resistant cells. (A) Drug‐naïve (naïve) or PM‐resistant (resistant) cells were allowed to attach overnight in a 6‐well plate. Mitochondrial mass was measured by flow cytometric analysis after the cells were labeled with a fluorescent dye 10‐*N*‐nonyl‐acridine orange (NAO). A representative FACS histogram from three separate experiments is shown in the upper panel. Bar graph (lower panel; N, drug‐naïve; Res, PM‐resistant) represents the mean ± SD of three independent experiments (*P* < 0.0001, unpaired two‐tailed *t*‐test). (B) Quantitative real‐time PCR assay was performed from RNA extracted from drug‐naïve (N) and PM‐resistant cells (Res). The level of expression of the PGC‐1α mRNA was assessed and normalized by ẞ‐actin mRNA (upper panel). All the data were analyzed from four different biological replicates with mean ± SD (unpaired two‐tailed *t*‐test). In parallel, samples were prepared for western blot analysis and probed with PGC‐1α and ẞ‐actin antibodies (lower panel). A representative blot is shown here. (C, D) PM‐resistant (Res) and drug‐naïve (N) glioma cells exposed to panobinostat and marizomib (25 nm of each) for 48 h (ST, short‐term). After harvesting, cellular ATP (C) or NAD^+^ content (D) was measured as described in the Section 2. Graph represents the data acquired from three independent experiments for each cell type (mean ± SD). Statistical significance was assessed with Tukey's ANOVA (C, D, ns, nonsignificant, *P < 0.05, **P < 0.005, ***P < 0.0005, and ****P < 0.0001).

Based on the observation that increased glycolytic and TCA cycle intermediates contributed to the observed resistance in glioma, we hypothesized that increased mitochondrial mass would directly correlate with drug resistance [77]. To address this issue, we examined mitochondrial mass using 10‐*N*‐nonyl‐acridine orange (NAO), a metachromatic dye that binds to the mitochondrial‐specific phospholipid, cardiolipin, where increased fluorescence intensity signal correlates well with increased mitochondrial content [78] regardless of their energetic state [79]. As shown in Fig. 4A, we noticed a significant increase in the relative intensity of the fluorescent dye in the resistant cells compared with drug‐naïve counterparts. Then, we looked at the expression level of PGC‐1α, an important coactivator that controls several aspects of mitochondrial function, including fuel intake, fatty‐acid oxidation, increased oxygen consumption, and mitochondrial biogenesis [80]. As shown in Fig. 4B, PGC‐1α mRNA transcript (upper panel) and protein (lower panel) level were robustly increased in resistant compared with naive cells. Similarly, since cellular ATP depletion is another marker of impaired mitochondrial bioenergetics, we examined the ATP content and noticed a significant depletion of intracellular ATP levels after short‐term treatment of cells with the combination of panobinostat and marizomib, which was reversed back to normal levels in the resistant cells (Fig. 4C), suggesting that the increased number of mitochondria and electron transport chain (ETC) activity may provide a highly efficient route for resistant cells to generate ATP.

NAD^+^ mediates several metabolic processes that are implicated in drug and radiation resistance in glioma [39, 81, 82]. Recently, using differential gene expression analysis, we identified the upregulation of quinolinic acid phosphoribosyltransferase (QPRT), an enzyme involved in the NAD^+^ biosynthesis in panobinostat and bortezomib‐resistant glioma cells [17]. Because continuous replenishment of NAD^+^ promotes the proliferation and survival of cancer cells [83], we next set out to determine the impact of panobinostat and marizomib on the NAD^+^ content in drug‐naïve and resistant glioma cell lines [39]. As shown in Fig. 4D, similar to our prior data with a related Pi [17], endogenous NAD^+^ level was barely detectable in the short‐term treated cells. By contrast, significant upregulation of NAD^+^ content was observed in the resistant counterparts. Because NAD^+^ coenzymes are critical for biosynthetic, bioenergetic, and ROS‐detoxifying processes on which normal and tumor cells depend [84, 85], we then examined nicotinic acid mononucleotide (NaMN), a precursor of coenzyme NAD^+^ (QPRT converts quinolinic acid to NaMN), nicotinic acid adenine dinucleotide (NaAD) and second messengers cADPR and ADPR. NAD^+^ is the substrate for the synthesis of these second messengers that are involved in the regulation of cellular Ca^2+^ homoeostasis [85, 86]. Indeed, quantitative targeted NAD metabolomics indicated that NaMn, NaAD, cADPR, and ADPR are all increased in concentration in PM‐resistant cells (Fig. S2E), suggesting that upregulation of ATP and NAD^+^ pathways may be necessary to meet the energy demand for cell survival and growth.

### 3.5 Targeting mitochondrial energetics overcomes PM resistance in glioma both *in vitro* and *in vivo*

**Fig. 5:**
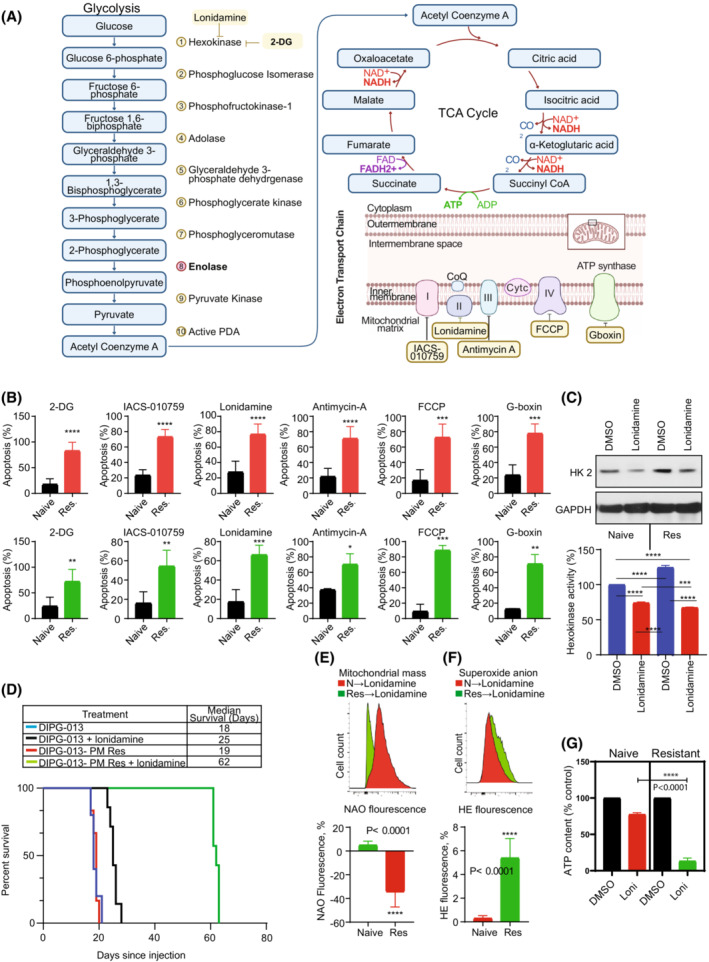
Targeting mitochondrial energetics overcomes panobinostat and marizomib‐induced drug resistance in glioma. (A) Graphical representation of therapeutic agents for targeting glycolysis and OXPHOS. Figure created using BioRender.com. (B) Drug‐naïve (Naïve) or PM‐resistant cells (Res) were seeded in 6‐well plates at the density of 1.5 × 10^5^ per well in 3 mL of media (DIPG‐013 upper panel; U87, lower panel). On the following day, cells were treated with either 2‐DG (10 mm), IACS‐010759 (10 μm), lonidamine (200 μm), antimycin A (10 μm), FCCP (25 μm) or Gboxin (10 μm), inhibitors of glycolysis or different component of the electron transport chain (ETC). After 3 days of incubation, apoptosis was analyzed by Annexin assay using flow cytometry. The results represent the mean of three independent experiments. Error bars indicate ± SD (unpaired two‐tailed *t*‐test; *P < 0.01, **P < 0.005; ***P < 0.0005; ****P < 0.0001). (C) Drug‐naïve (Naïve) or PM‐resistant (Res) DIPG‐013 cells were treated with lonidamine (100 μm) for 72 h. Control cells received vehicle (DMSO). Cell extracts were prepared using hexokinase assay buffer (provided in the hexokinase enzyme activity assay kit as described in the Section 2). Twenty micrograms of protein were loaded on a sodium dodecyl sulfate‐polyacrylamide gel and probed with the indicated antibodies by western blotting. Fifty micrograms of protein were used to measure hexokinase enzyme activity. These experiments were performed three times, and a representative blot (upper panel) and the change in hexokinase enzyme activity relative to drug‐naïve control are presented in the lower panel. Data represent the mean ± SD from three independent experiments. Statistical significance was assessed with Tukey's ANOVA (***P < 0.0005, ****P < 0.0001). (D) Kaplan–Meier analysis of *in vivo* models of drug‐naïve or PM‐resistant DIPG‐013 tumors treated with either vehicle control or lonidamine. DIPG‐013‐naïve models treated with vehicle control (*n* = 5) had a median survival of 18 days, while DIPG‐013‐naïve cells treated with lonidamine (*n* = 7) showed a significantly increased median survival of 25 days (*P* = 0.0003). PM‐resistant DIPG‐013 models (*n* = 6) showed a median survival of 19 days, while the lonidamine‐treated group (*n* = 7) showed a significant increase in median survival of 62 days (*P* = 0.0003). No difference in survival was noted between DIPG‐013‐naïve vs. ‐resistant cells. (E–G) Drug‐naïve (N) or PM‐resistant (Res) DIPG‐013 cells were allowed to attach overnight in a 6‐well plate. On the following day, cells were treated with lonidamine (100 μm). Mitochondrial mass (E) and superoxide anion (F) content was measured by flow cytometric analysis. The representative FACS histogram is shown in the upper panel. Bar graph (bottom panel) represents the mean of three independent experiments ± standard deviation (E, F, unpaired two‐tailed *t*‐test; ****P < 0.0001). (G) Cellular ATP content was assessed as described in the Section 2. Data represent the mean ± SD from three independent experiments. Statistical significance was assessed with Tukey's ANOVA (****P < 0.0001). mean ± SD.

To test the hypothesis that targeting mitochondrial energetics could reverse panobinostat and marizomib‐induced resistance in glioma both *in vitro* and *in vivo*, we examined the impact on the resistance of several inhibitors of oxidative phosphorylation (OXPHOS) and the electron transport chain (ETC). Cells were treated with 2‐DG, an antidiabetic agent that inhibits glycolysis, and with mitochondrial respiratory chain inhibitors IACS‐010759 (inhibitor of complex I), lonidamine (inhibitor of complex II and hexokinase II), antimycin A (inhibitor of complex III), FCCP (mitochondrial uncoupler), and Gboxin (ATP synthase inhibitor; Fig. 5A). PM‐resistant DIPG‐013 (Fig. 5B, upper panel) and U87 (Fig. 5B, lower panel) display a substantial sensitivity to each agent *in vitro*.

We then investigated whether the observed increase in cell death *in vitro* would translate into an overall survival benefit in the DIPG‐013 (H3.3K27M‐mutant) animal model. We selected lonidamine since it has previously been safely applied *in vivo* and has been shown to interfere with hexokinase activity and mitochondrial energy metabolism by inhibiting glycolysis, triggering dissipation of the mitochondrial transmembrane potential, and increasing ROS generation [35, 87, 88, 89]. To first directly test the hypothesis that lonidamine inhibits hexokinase (HK) protein expression and activity, cell extracts prepared were subjected to western blot analysis and HK enzyme activity assay. We confirmed that lonidamine inhibited hexokinase 2 protein expression *in vitro* (Fig. 5C, upper panel) and also the enzyme activity (Fig. 5C, lower panel). While PM‐resistant cells stimulated HK activity (28% increase vs. drug‐naïve control cells), the level of inhibition induced by lonidamine was more pronounced in PM‐resistant cells (lonidamine treatment inhibited 45% of the HK enzyme activity of PM‐resistant cells compared with 25% to their lonidamine‐treated drug‐naïve cells, Fig. 5C, lower panel).

To validate this hypothesis *in vivo*, we completed stereotactic intracranial injection of DIPG‐013‐naïve or ‐resistant cells into the midbrain of NOD‐SCID mice, producing two DIPG models. Of note, in our previous study [17], to mimic the clinical scenario, cells were allowed to recover in drug‐free media where we observed the persistence of resistance phenotype for at least 30 days after HDACi/Pi drug removal. Both naïve and resistant models were treated with either vehicle control (5% DMSO in Tris/glycine buffer) or lonidamine (50 mg·kg^−1^) via intraperitoneal injection with 2 cycles of 5 doses of lonidamine consisting of 1 dose per day, with 2 days intervening between cycle. Kaplan–Meier analysis indicated that lonidamine significantly increased the survival distribution of DIPG‐013‐naïve mice, with a median survival increase from 18 to 25 days (*P* = 0.0003). Additionally, survival significantly increased in PM‐resistant models with lonidamine treatment, where the median lifespan increased from 19 to 62 days (*P* = 0.0003; Fig. 5D).

To ascertain the functional effect of lonidamine, we examined mitochondrial mass, ROS generation, and ATP content. We observed a significant loss of mitochondrial mass in response to lonidamine in PM‐resistant cells compared with lonidamine‐treated control cells (Fig. 5E). Also, treatment with lonidamine increased ROS generation (Fig. 5F) of PM‐resistant cells and as expected, significantly decreased the amount of ATP content of PM‐resistant cells compared with naïve counterparts treated with lonidamine (Fig. 5G), suggesting that targeting mitochondrial energetics is a promising therapeutic strategy for counteracting PM‐induced resistance in glioma.

## 4 Discussion

Although FDA‐approved proteasome inhibitors, bortezomib and carfilzomib, showed a limited efficacy in the preclinical models due to poor tumor penetration, marizomib, an orally active, small molecule proteasome inhibitor gained interest because of its ability to cross the blood–brain barrier and exert significant antitumor activity in glioma as a single agent and/or in combination therapies [90]. By quantitative high‐throughput screens (HTS) of 2706 approved investigational drugs and combination assessments encompassing 9195 drug–drug examinations, Lin et al. [20] demonstrated that combining marizomib and panobinostat as a promising therapeutic approach in DMG providing a rationale for evaluation in patients with brain malignancies. Consistent with our previous findings [17, 27], the combination of marizomib and panobinostat substantially improved the therapeutic efficacy in both pediatric and adult glioma cell lines in a synergistic manner. However, despite recent advances in our understanding of the molecular underpinnings of glioma during the past decades, resistance to chemotherapeutic agents and/or novel targeted signaling inhibitors continues to be a major problem in disease management. The present study indicates that the activity of panobinostat and marizomib dramatically potentiated cell death via increasing mitochondrial injury, inhibition of ATP generation, and caspase activation in pediatric and adult HGG and DIPG cell lines, despite their molecular differences, providing the rationale for clinical trials to address this devastating disease [20, 91, 92]. Although the potentiation of anticancer effects by the combination of panobinostat and marizomib has been reported previously [20], little attention has been directed toward understanding mechanisms of resistance to this class of inhibitors [28], which is of vital importance given the propensity of these tumors to rapidly fail initial therapies. We selected panobinostat and marizomib over vorinostat and bortezomib for our *in vitro* and *in vivo* studies because of their superior blood–brain barrier penetrance [25, 93]. Here, we identified and characterized a critical resistance mechanism to this combination of agents, with the goal of leveraging the insights obtained from this study to develop strategies for counteracting this process.

To determine the mechanism of panobinostat and marizomib‐induced cytotoxicity, we performed several *in vitro* assays, including staining with annexin V–fluorescein isothiocyanate to quantify apoptosis, assessment of cell cycle profiles and mitochondrial membrane potential, and analysis of ROS, NAD^+^ and ATP content. In contrast to normal conditions where the mitochondrial inner membrane is practically impermeable [94], cotreatment with panobinostat and marizomib led to the loss of mitochondrial membrane potential and increased generation of ROS. Then, we selected for PM‐resistant adult, pediatric and DIPG cell lines that were indistinguishable from their drug‐naïve counterparts with respect to morphology, cell cycle profile, and colony‐forming ability. In these resistant cells, we demonstrated a significant increase in mitochondrial mass and transcription and translation of PGC‐1α, a known mediator of mitochondrial biogenesis in the resistant cells, suggesting high mitochondrial mass was associated with panobinostat‐marizomib resistance, supporting the idea that elevated mitochondrial function (i.e., generation of ATP among other energy metabolites) may be essential for the resistant cells to survive and proliferate [95, 96]. Consistent with our recent observation [17], the NAD^+^ levels in all resistant cell lines were elevated in comparison with short‐term treated counterparts raising the possibility that resistant cells efficiently replenish the supply of NAD^+^ to provide a survival advantage and may create new therapeutic vulnerabilities [17].

Liu et al. [70] demonstrated that ENO2 promoted glycolysis and cell growth, which were reduced when ENO2 was silenced using shRNA. They also showed that the ENO2 mRNA expression was increased when patients suffered a relapse in ALL, suggesting that ENO2 may be a biological marker for monitoring chemotherapeutic efficacy, supporting the idea that upregulation of the glycolytic pathway, as noted in our resistant cells, may reflect adaptation to cellular stresses. We also demonstrated a significant increase in PDH activity in the resistant cells, which is the first step in the TCA cycle. Similarly, an increased amount of cis‐aconitate, an intermediate in the isomerization of citrate to isocitrate in the second step of the citric acid cycle, was evident in the resistant cells. Likewise, other members of the TCA cycle, including succinate and fumarate were increased in the setting of resistance. We observed a modest increase in malate in the resistant cells after a significant reduction after short‐term treatment with the inhibitors, implicating this pathway as an overall resistance mechanism. The nucleoside inosine that acts as a molecular messenger in cell signaling pathways that plays an important role in purine biosynthesis and gene translation showed a profound increase in resistant cells, suggesting the importance of secondary metabolites in drug resistance [97, 98]. Similar results were observed with the nucleoside cytidine, a pyrimidine component of RNA. Together, these observations suggest that PM‐resistant glioma cells rely significantly on glycolysis, TCA cycle, and nucleoside biosynthesis to counteract drug response.

To validate the functional role of these pathways in resistance, we examined the impact of inhibitors of glycolysis and electron transport chain complexes on PM‐resistant vs. drug‐naïve cells. We demonstrated that treatment with 2‐deoxyglucose (2‐DG), a glucose analog that inhibits glycolysis, induced a significant reduction in cell viability of PM‐resistant cells compared with drug‐naïve cells. Similarly, treating resistant cells with lonidamine, an inhibitor of hexokinase and complex II (of electron transport chain) [31, 32], resulted in a significant reduction in hexokinase expression, hexokinase enzyme activity, mitochondrial mass, increased ROS generation and ATP depletion, and increased cell death. This was consistent with an earlier report by Miccoli et al. [99] that demonstrated that lonidamine enhanced cell cycle arrest and altered glioma metabolism *in vitro* and *in vivo*. Our observation that sensitivity to this agent was dramatically enhanced in resistant cells compared with control, highlights the potential for glioma bioenergetic dependency on glycolysis as cells become increasingly resistant to constitute an actionable therapeutic vulnerability that has heretofore not been exploited.

Then, to test this hypothesis in the *in vivo* context, we chose lonidamine because of its impressive therapeutic activity in our *in vitro* studies as an inhibitor of both glycolysis and the electron transport chain, and its track record of tolerability in clinical therapeutics [31, 100, 101, 102, 103, 104, 105, 106, 107, 108, 109]. Our results showed a significant extension of survival in naïve cells but an even more dramatic enhancement of survival in PM‐resistant tumors, with more than a 3‐fold prolongation of median survival. This fits with our *in vitro* observations of loss of mitochondrial membrane potential, increased generation of ROS, loss of mitochondrial mass, and cell viability that were particularly dramatic in resistant cells.

## 5 Conclusions

The results suggest that physiologically relevant concentrations of panobinostat and marizomib have significant pro‐apoptotic activity in pHGG and aHGG cell lines. However, the challenge presented is neoplastic cells acquire resistance to this combination therapy at clinically achievable doses, limiting the effectiveness of these therapeutic agents. We provide evidence that the emergence of resistance to panobinostat and marizomib is generated due to the highly integrated TCA cycle metabolic network, driving the resistant cells to efficiently replenish the supply of ATP content for survival and growth. In summary, these observations support a critical role for the upregulation of glycolysis and energy metabolism as a mechanism for glioma treatment resistance, which provides a targetable metabolic vulnerability that can be exploited by agents such as lonidamine to enhance glioma cell killing and prolong survival and represents a novel therapeutic strategy that warrants further exploration.

## Conflict of interest

The authors declare no conflict of interest.

## Author contributions

DRP, SA, and IFP conceptualized and designed the study. EPJ, MCR, TAG, MEH, TAM, SJM, UO, DM, and DRP performed the experiments. DRP, EPJ, MCR, TAG, MEH, BJG, SMC, SGW, UO, DM, RD, AM, SA, CB, and IFP analyzed the data. SA, DRP, and IFP supervised the study. All authors participated in data interpretation and manuscript writing, review, and editing. All authors read and approved the final manuscript.

### Peer review

The peer review history for this article is available at https://www.webofscience.com/api/gateway/wos/peer‐review/10.1002/1878‐0261.13427.

## Supporting information


**Fig. S1.** Enhanced glycolysis and the mitochondrial TCA cycle metabolites are associated with panobinostat‐ and marizomib‐induced resistance.

**Fig. S2.** Quantitative analysis of nucleosides and NAD^+^ metabolites.

**Table S1.** Interaction landscape of the combination of panobinostat and marizomib for KNS42, SJG2, SF8628, U87, LNZ308, and T98G cell lines.

**Table S2.** Differential gene expression between drug‐naïve vs. PM‐resistant DIPG‐007 and DIPG‐013 cell lines.

**Data S1.** Figure Legends.

**Data S2.** Table Legends.

## Data Availability

No datasets were generated or submitted related to this paper that is available in a public database.
