# Supplementary material for: Targeting mitochondrial energetics reverses panobinostat‐ and marizomib‐induced resistance in pediatric and adult high‐grade gliomas
Source: Mol Oncol. 2023 May 12;17(9):1821–43. doi: 10.1002/1878-0261.13427 (PMC10483615; doi:10.1002/1878-0261.13427)
Supplement: Supplementary file 1 — Fig. S1. Enhanced glycolysis and the mitochondrial TCA cycle metabolites are associated with panobinostat‐ and marizomib‐induced resistance. [file MOL2-17-1821-s001.pdf]

Fig. S1

S1A

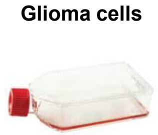

- ❑ Incubate cells in a cocktail containing panobinostat and marizomib (2.5 nM each) for 3-5 days
- ❑ Isolate live cells (Q3) by flow cytometry
- ❑ Transfer cells to a new flask containing 2X inhibitor concentration
- ❑ Incubate for 3-5 days
- ❑ Isolate live cells by flow cytometry
- ❑ Repeat the cycle until it reaches panobinostat+ marizomib 25 nM each

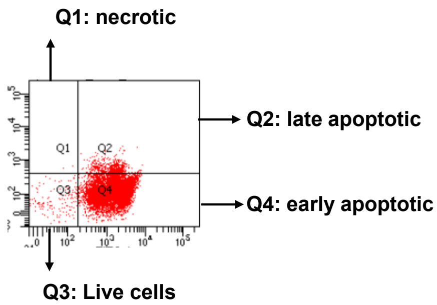

S1B

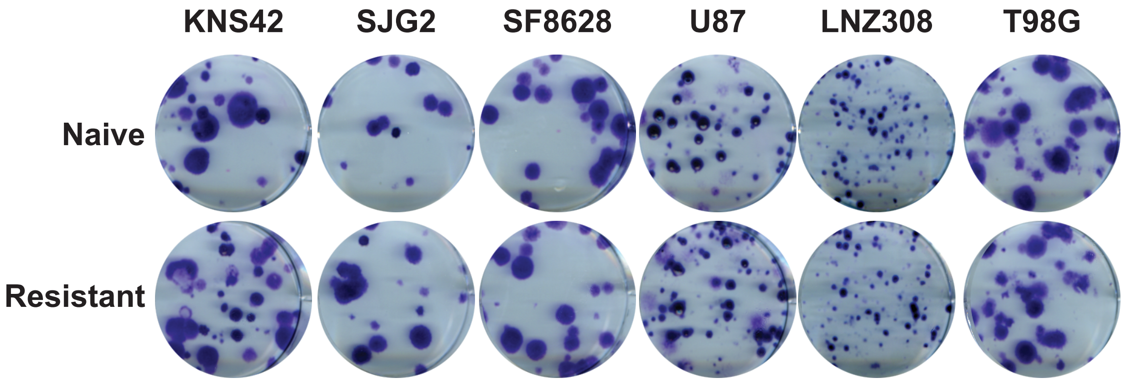

S1C

Cell line	Number of days to generate resistant population	Approximate number of passages required to generate resistance
**U87**	65	25
**LNZ308**	70	30
**T98G**	55	25
**KNS42**	90	40
**SJG2**	80	40
**SF8628**	110	60
**DIPG-007**	90	40
**DIPG-013**	90	40
