# Supplementary figures and images for: Targeting mitochondrial energetics reverses panobinostat‐ and marizomib‐induced resistance in pediatric and adult high‐grade gliomas
Source: Mol Oncol. 2023 May 12;17(9):1821–43. doi: 10.1002/1878-0261.13427 (PMC10483615; doi:10.1002/1878-0261.13427)

Fig. S2

A

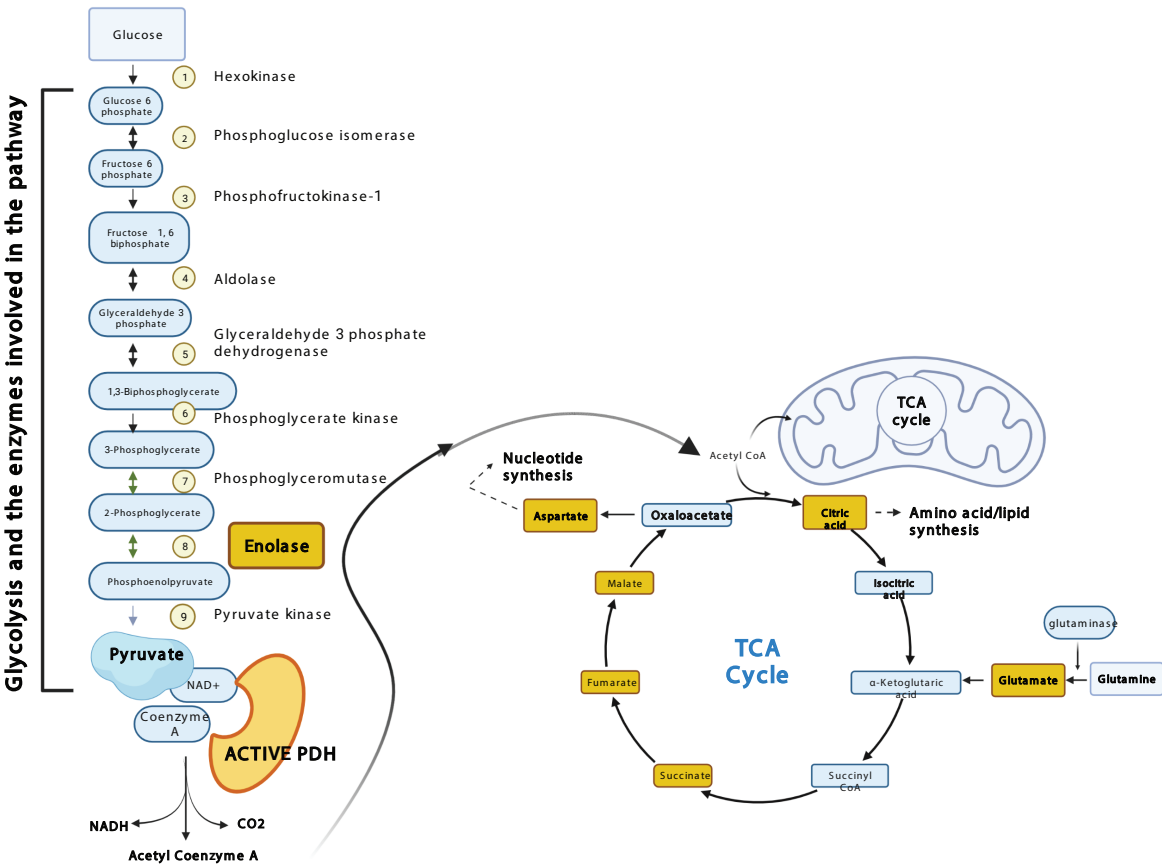

B

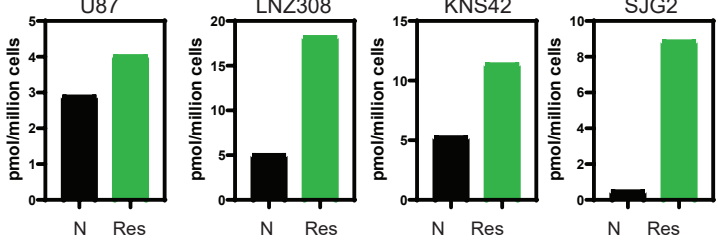

C

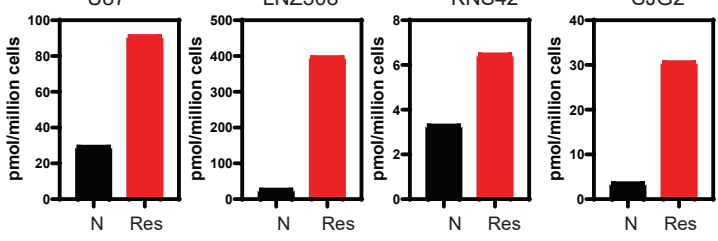

D

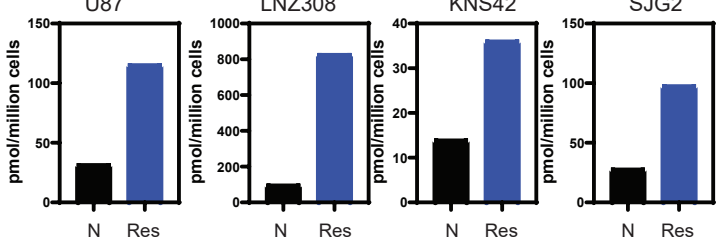

E

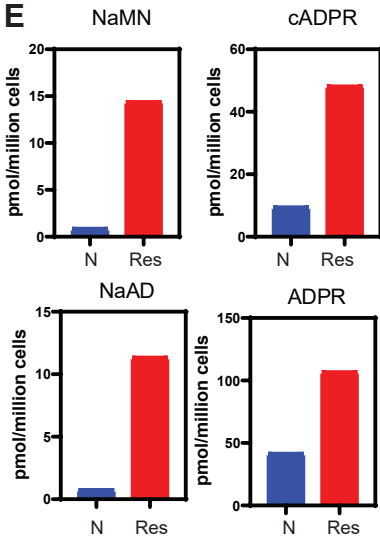

Supplement: Supplementary file 2 — Fig. S2. Quantitative analysis of nucleosides and NAD+ metabolites. [file MOL2-17-1821-s003.pdf]
