# Supplementary material for: Targeting mitochondrial energetics reverses panobinostat‐ and marizomib‐induced resistance in pediatric and adult high‐grade gliomas
Source: Mol Oncol. 2023 May 12;17(9):1821–43. doi: 10.1002/1878-0261.13427 (PMC10483615; doi:10.1002/1878-0261.13427)
Supplement: Supplementary file 5 — Data S1. Figure Legends. [file MOL2-17-1821-s004.docx]

**Supplementary Figure Legends**

**Fig. S1.**

**Enhanced glycolysis and the mitochondrial TCA cycle metabolites are associated with panobinostat and marizomib-induced resistance. A.** Pediatric and adult HGG and DIPG cells were exposed to the combination of panobinostat and marizomib. Each original parental cell lines were treated with the combination of panobinostat and marizomib (2.5 nM each). Drug-naïve control cells received an equal amount of vehicle (DMSO). Viable cells were isolated, washed with PBS and cultured in growth media containing double the concentration of inhibitors. This process was repeated until the cells grew at a comparable rate in the presence of panobinostat and marizomib (25 nM each). **B**. Drug naïve (seeded and cultured in complete growth media) of panobinostat and marizomib-resistant (seeded and cultured in the media containing panobinostat and marizomib, 25 nM each) cells were seeded in a six-well plate (300 cells per well). Clonogenic assay was performed as described in the Materials and Methods. These experiments were performed at least three times, and a representative image is presented. **C**. Number of passages and duration in days required to develop resistant cell lines used in this study. Cell lines were treated with the combination of panobinostat and marizomib (2.5 nM each). Drug-naïve control cells received an equal amount of vehicle (DMSO). Viable cells were isolated, washed with PBS and cultured in growth media containing double the concentration of inhibitors. This process was repeated until the cells grew at a comparable rate in the presence panobinostat and marizomib (25 nM each). The resistance-development period was about 2-4 months. During this time, cell viability, clonogenic potential and cell cycle profile was assessed between drug-naïve control and resistant cells to determine changes in sensitivity to the inhibitors.

**Fig. S2.**

**Quantitative analysis of nucleosides and NAD+ metabolites. A.** Schematic depicting the metabolites and enzymes involved in glycolysis and the TCA Cycle and the contribution of enolase-2 (ENO2) as the penultimate step in the glycolytic process. Figure created using BioRender.com. Cellular (N, naïve or resistant, Res, PM-resistant) cytidine (**B**), inosine (**C**) and IMP (**D**) were measured using MRM LC-MS as described in the methods. **E**, Analysis with LC/MS of drug-naïve (N) and PM-resistant (Res) SJG2 cells, showing upregulation of NAD+ pathway components.
