# Supplementary material for: Targeting mitochondrial energetics reverses panobinostat‐ and marizomib‐induced resistance in pediatric and adult high‐grade gliomas
Source: Mol Oncol. 2023 May 12;17(9):1821–43. doi: 10.1002/1878-0261.13427 (PMC10483615; doi:10.1002/1878-0261.13427)
Supplement: Supplementary file 6 — Data S2. Table Legends. [file MOL2-17-1821-s005.docx]

**Supplementary Table 1.**

**Interaction landscape of the combination of panobinostat and marizomib for KNS42, SJG2, SF8628, U87, LNZ308 and T98G cell lines.**

**Supplementary Table 2.**

**Differential Gene Expression between drug naïve versus PM-resistant DIPG-007 and DIPG-013 cell lines.**
